# Hepatic mitochondrial dysfunction is a feature of Glycogen Storage Disease Type Ia (GSDIa)

**DOI:** 10.1038/srep44408

**Published:** 2017-03-20

**Authors:** Benjamin L. Farah, Rohit A. Sinha, Yajun Wu, Brijesh K. Singh, Andrea Lim, Masahiro Hirayama, Dustin J. Landau, Boon Huat Bay, Dwight D. Koeberl, Paul M. Yen

**Affiliations:** 1 Cardiovascular and Metabolic Disorders Program, Duke-NUS Medical School Singapore, Singapore; 2 Department of Anatomy, Yong Loo Lin School of Medicine, National University of Singapore, Singapore; 3 Faculty of Medicine, Tohoku University, Sendai, Japan; 4 Department of Molecular Genetics and Microbiology, Duke University, Durham, NC, USA; 5 Division of Medical Genetics, Department of Pediatrics, Duke University Medical Center, Durham, NC, USA; 6 Duke Molecular Physiology Institute, Duke University School of Medicine, Durham, NC, USA

## Abstract

Glycogen storage disease type Ia (GSDIa, von Gierke disease) is the most common glycogen storage disorder. It is caused by the deficiency of glucose-6-phosphatase, an enzyme which catalyses the final step of gluconeogenesis and glycogenolysis. Clinically, GSDIa is characterized by fasting hypoglycaemia and hepatic glycogen and triglyceride overaccumulation. The latter leads to steatohepatitis, cirrhosis, and the formation of hepatic adenomas and carcinomas. Currently, little is known about the function of various organelles and their impact on metabolism in GSDIa. Accordingly, we investigated mitochondrial function in cell culture and mouse models of GSDIa. We found impairments in oxidative phosphorylation and changes in TCA cycle metabolites, as well as decreased mitochondrial membrane potential and deranged mitochondrial ultra-structure in these model systems. Mitochondrial content also was decreased, likely secondary to decreased mitochondrial biogenesis. These deleterious effects culminated in the activation of the mitochondrial apoptosis pathway. Taken together, our results demonstrate a role for mitochondrial dysfunction in the pathogenesis of GSDIa, and identify a new potential target for the treatment of this disease. They also provide new insight into the role of carbohydrate overload on mitochondrial function in other hepatic diseases, such as non-alcoholic fatty liver disease.

Glycogen Storage Disease Type Ia (GSDIa, von Gierke disease), the most common glycogen storage disorders, affects about 1 out of every 100,000 live births^1^. As originally described by von Gierke, there is a marked increase in the size of both the liver and kidney due to glycogen accumulation^2^. This disease is caused by a loss of function in glucose-6-phosphatase α (G6Pase α, G6PC), an ER-resident enzyme that is expressed primarily in the kidney and liver. This enzyme catalyses the hydrolysis of glucose-6-phosphate (G6P) to form glucose, which is the common final step in both glycogenolysis and gluconeogenesis^3^. The loss of function of this enzyme results in an accumulation of G6P, leading to increased glycogen and lipid synthesis, lactate production, and a concomitant decrease in free glucose production^4^,^5^,^6^,^7^. Patients with GSDIa usually present in infancy with severe hypoglycaemia^6^; other serum abnormalities include increased levels of uric acid, lactic acid, triglycerides as well as VLDL^8^. Patients often develop gout and renal failure^9^, and universally develop steatohepatitis that culminates in cirrhosis, hepatic adenomas and hepatomas^3^,^10^,^11^. Current treatments are limited to dietary therapy to maintain blood glucose levels^3^, and liver transplant, which can be curative^12^. As a result, death occurs prematurely in affected patients, usually in the third or fourth decade of life^13^.

The metabolic derangements seen in GSDIa previously were thought to be due solely to the increased levels of various metabolites such as glucose-6-phosphate and acetyl-CoA “pushing” biochemical pathways towards glycogen, nucleotide, and lipid synthesis. However, other metabolic and cellular mechanisms may contribute to the pathogenesis of this disease. For example, previous work has shown that activation of the ChREBP pathway^14^, and increased hepatocellular apoptosis^15^, are both responsible for some of the features of this disease. Furthermore, our group has recently shown that deranged autophagy plays a key role in the pathogenesis of GSDIa^16^. However, little is currently known about the function of various organelles and their impact on metabolism in GSDIa.

Mitochondria provide most of the cell’s oxidative metabolism and energy generation, and are the sites where fatty acid β-oxidation, the tri-carboxylic cycle (TCA cycle), and oxidative phosphorylation occur^17^. Primary dysfunction of mitochondria leads to progressive muscular and neurological degeneration^18^. Beyond these primary mitochondrial disorders, mitochondrial dysfunction may contribute to the development of other metabolic diseases, such as Type II diabetes mellitus^19^. Besides its crucial role in energy production, mitochondria also integrate other important cellular signalling pathways^20^, notably apoptosis signalling^21^. In the intrinsic apoptosis pathway, damaged mitochondria are no longer able to maintain the proper electrical potential across their membranes, resulting in the release of a mitochondrial protein, cytochrome c, into the cytosol^21^,^22^. Cytochrome c then binds to a complex of proteins which in turn activate downstream effectors of apoptosis^21^,^23^,^24^.

Since mitochondria play a key role in both cellular energy metabolism and apoptosis, it is possible that the function of this organelle is impaired in GSDIa. However, the role of mitochondria in this disease has not been well characterized. Previous work showed that there is a decrease in mitochondrial number with no change in mitochondrial volume in the livers of young GSDIa patients^25^. Furthermore, neutrophils taken from a mouse model of a very similar disorder, GSDIb, showed an increase in mitochondrial-mediated apoptosis^26^. Accordingly, we undertook an investigation into the function of mitochondria in GSDIa, to understand their potential role in the pathogenesis of this disease. Furthermore, we hypothesized that mitochondrial dysfunction may play a role in the increased apoptosis observed in this disorder^15^. Analysing both cell culture and mouse models of GSDIa, we found that oxidative metabolism was impaired in this disease, and was associated with changes in the levels of tri-carboxylic acid cycle (TCA cycle) intermediates, derangements in mitochondrial structure, and a decrease in mitochondrial number. These changes culminate in an activation of the mitochondrial apoptosis cascade. Our findings shed new light on the role of mitochondrial dysfunction in the pathogenesis of GSDIa and carbohydrate excess, and suggest new potential molecular targets for their treatment.

## Materials and Methods

### Study Approval

All animal studies performed were approved by the Duke University Institutional Animal Care and Use Committee (IACUC) under the protocols A231-11-09 and A083-11-04. All reasonable and necessary steps to reduce animal suffering were undertaken, and all animals received care as per the criteria outlined in NIH publication 86-23. The authors also confirm that all experiments were performed in accordance with relevant guidelines and regulations.

### Statistics

Cell culture experiments were performed in triplicates and repeated 3 independent times using matched controls. All statistical calculations were carried out using Microsoft Excel. For all parts, statistical significance was calculated using Student’s t-test, and p < 0.05 was taken to be significant. Results were expressed as mean + SEM.

### Reagents

Glutaraldehyde (G7651), para-formaldehyde (158127), dexamethasone (D4902), foetal bovine serum (12003 C), CCCP (C2759), DCFDA (35845), tBHP (458139), antibody recognizing PGC1α (SAB2500781), and oligionucleotide primers were from Sigma-Aldrich. Mitochondrial fractionation kit (89874), TMRE (T-669), and Pierce BCA Assay Kit (23225) were purchased from Thermo Scientific. DMEM/F12 1:1 (11320), Insulin-Transferin-Selenium (ITS) (41400), Penicillin-Streptomycin (15140), RNAiMax (13778150), and siRNAs targeting mouse G6PC (MSS204485, MSS204486, MSS204487) came from Life Technologies. XF Cell Mitostress Kit (101706-100) and XF assay media (102365) were purchased from Seahorse Bioscience. Antibodies recognizing α-Tubulin (2144), cleaved caspase 3 (9661), cytochrome C (4280), VDAC (4661), TFAM (8076), NRF1 (12381), cleaved caspase 9 (9509), acetylated lysine (9441), COXIV (4850), PHB1 (2426), and SDHA (11998) were all from Cell Signalling Technologies. The antibody recognizing G6PC (83690) came from Abcam. Antibodies recognizing TOM20 (sc-11415), β-actin (sc-8432) as well as HRP conjugated secondary antibodies recognizing mouse (sc-2954), goat (sc-2953) and rabbit (sc-2955) IgG were purchased from SantaCruz Biotechnologies.

### Cell Culture

AML-12 (CRL-2254) cells were purchased from ATCC and maintained at 37 °C in DMEM/F12 1:1 containing 10% FBS, 1x ITS, 10 nM dexamethasone and 1x penicillin/streptomycin in a 5% CO_2_ atmosphere. Cells were placed in DMEM/F12 1:1 without serum or supplements for 24 h prior to passaging or knock-down. For all siRNA experiments, cells were transfected using RNAiMAX following the manufacturer’s protocol for reverse-transfection. Unless otherwise noted, assays were performed 96 hrs. after siRNA transfection. Knock-down efficiency was ascertained by western blotting for G6PC, a typical knock-down result is shown in Suppl. Fig. 1.

### Western Blotting

Cultured cells lysed in mammalian lysis buffer (Sigma-Aldrich), with appropriate phosphatase and protease inhibitors, followed by scraping. Tissues were homogenized by MagNA Lyser beads (Roche) in the same buffer. Protein concentration was determined using the BCA Kit (Bio-Rad), and protein was stored at −80 °C. Prior to western blotting, Laemmli sample buffer was added to the samples (250 mmol/l Tris, pH 7.4, 2% w/v SDS, 25% v/v glycerol, 10% v/v 2-mercaptoethanol, and 0.01% w/v bromophenol blue), and the samples were then heated to 105 °C for 5 minutes, kept at 4 °C for 10 minutes, followed by separation on a SDS-polyacrylamide gel. Proteins were then transferred to a polyvinylidine difluoride membrane (Bio-Rad) in transfer buffer (25 mmol/l Tris, pH 8.8, 192 mmol/l glycine, and 10% v/v methanol). All washing, blocking and antibody solutions were prepared in PBS with 0.1% Tween-20 (PBST). Membranes were blocked for one hour in 5% milk, followed by overnight incubation at 4 °C with primary antibodies in 1% bovine serum albumin. Membranes were washed thrice, followed by secondary antibody incubation at room temperature for 1 hr. in 1% bovine serum albumin, followed by 3 more washes in PBST, and placed in PBS. Blots were probed using an enhanced chemiluminescence system (GE Healthcare) on a GelDoc imager (Bio-Rad). Densitometry was performed using ImageJ software (NIH).

### Mitochondrial Oximetry

Oxygen consumption was measured on a Seahorse XF24 analyser at 37 °C. 48 hours before assay, 40,000 cells of siG6PC or siNeg treated AML-12 cells were seeded in a Seahorse XF24 analyser plate. The following steps were all performed as per the manufacturer’s recommendations. On day of assay, media was replaced with seahorse assay media. Oxygen consumption rate was measured in the basal state (basal OCR), following oligomycin treatment, and following FCCP addition. ATP turnover was calculated by dividing the absolute value of the difference of oligomycin treated OCR and basal OCR by the basal OCR. The oxygen consumption rate following FCCP addition was defined as maximal respiratory capacity. The spare mitochondrial capacity was calculated by subtracting the basal OCR from the OCR following FCCP addition. Following the assay, cells were lysed, and protein content taken for normalization.

### mRNA isolation and quantitative real-time PCR

RNA was isolated from cells by the Invitek Mini Kit (Invitek) or from tissues using TriZOL (Sigma-Aldrich), followed by clean-up on the Invitek Mini Kit. 1 μg RNA was reverse-transcribed using the iScript Select cDNA Synthesis Kit (Bio-Rad). Quantitative Real-Time PCR was performed using the QuantiTect SYBR Green Kit (QIAGEN). RNA Polymerase II (PolII) expression was used for normalization, while fold change was calculated using 2^−ΔΔCt^. Primer sequences are available in Table 1.

### Electron Microscopy

Fresh tissue was placed in fixative (2% para-formaldehyde and 3% gluteraldehyde in pH 7.4 cacydolate buffer) and stored at 4 °C, whereas cultured cells were grown in glass chambers, and fixed overnight in fixative (2% para-formaldehyde and 3% gluteraldehyde in pH 7.4 phosphate buffer). Samples were washed once in PBS, followed by post-fixation treatment with 1% osmium tetroxide. Samples were dehydrated with ascending concentrations of alcohol, followed by Araldite embedding. Ultrathin sections were cut then stained with uranyl acetate and lead citrate. Images were acquired on an Olympus EM208S transmission electron microscope.

### Mouse Experiments

Heterozygous G6PC^+/−^ mice were housed in the Duke Vivarium and bred to produce homozygous G6PC^−/−^ offspring. Genotype was confirmed by PCR analysis of tail DNA with primers within and flanking the neo gene insertion in the G6Pase gene as has been previously published^27^. Due to known difficulties in breeding these mice, the majority of mouse tissue used for the experiments in this work came from mice sacrificed previously^16^. Injections of 0.1 to 0.2 mL 10% dextrose subcutaneously were performed daily starting at 3 days of age and continued for 2–3 weeks or until sacrifice. Mice were sacrificed in the fed state by CO_2_ incubation at 10 days of age, and tissues were immediately harvested and either snap frozen in liquid nitrogen, or fixed in the electron microscopy fixative. Wild-type litter-mates of the same age were used as controls. Details regarding the metabolic parameters of the mice used in these experiments are presented in Table 2.

### Mitochondrial Fractionation

Mitochondrial fractionation was performed using Thermo Scientific Mitochondrial Fractionation kit according to the manufacturer’s instructions.

### Mitochondrial Membrane Potential Analysis

AML-12 cells were transfected with either siNeg or siG6PC on a black 96 well plate. 72 hours after knock-down, 80 μM CCCP in growth media was added to half of the siNeg cells for 10 minutes. Media for all of the cells was then replaced with media containing TMRE at a concentration of 400 nM for 20 minutes (with CCCP additionally added to the CCCP treated cells). The media was then removed, and replaced with 0.2% BSA in PBS. The emission at 590 nm was recorded following excitation at 520 nm on a fluorescent plate reader.

### Cellular ROS determination

AML-12 cells were grown and treated with siNeg or siG6PC on a black 96 well plate. At indicated times, cells were washed with PBS, and then 25 μM DCFDA in PBS was added to the cells for 45 minutes. Following staining, cells were incubated in media with/without 50 μM tBHP (positive control) for 15 minutes, then placed in PBS. The emission at 535 nm was recorded after excitation at 485 nm on a fluorescent plate reader.

### Metabolomics

Organic acids and amino-acids were extracted from 100 μL of liver homogenate using methanol and then derivatised to form butyl esters using 3 M HCl in butanol. Samples were then reconstituted in 80% aqueous methanol and 4 μL of this solution was injected into an Agilent SB-C8 column (12 × 50 vmm with 1.8 um particle size) for analysis. Mobile phase used was 80% methanol and 20% water, and flow rate was maintained at 0.4 ml/min for 2 min. Isocratic flow of 0.6 ml/min of 30% acetonitrile and 70% water with 0.1% formic acid was maintained for 5.5 min.

### Mitochondrial DNA content analysis

Cells were treated with siRNA against G6PC, and grown for 96 hours. Total DNA was extracted, and stored at −80 °C. ND1 gene was used as a marker for mitochondrial DNA content (Primers: F: 5′-CCCATTCGCGTTATTCTT-3′ R: 5′-AAGTTGATCGTAACGGAGC-3′), and Lpl gene was used as a marker for nuclear DNA content (Primers: F: 5′-GGATGGACGGTAAGAGTGATTC-3′ R: 5′-ATCCAAGGGTAGCAGACAGGT-3′). 10 ng of genomic DNA, 12.5 μL of SybrGreen (Qiagen), 25 μM of forward and reverse primers, and dH20 to a total volume of 25 μL was added to each quantitative-real time PCR reaction tube. Quantitative-real time PCR was performed with the following parameters (95 °C for 1 minute, followed by 40 cycles of 95 °C for 30 s, 62 °C for 30 s, and 72 °C for 30 s, with signal acquisition performed during this last step). Following amplification, the samples were heated to 95 °C, with the dissociation curve examined to confirm the specificity of the PCR reaction. The mtDNA content was calculated using 2^−ΔΔCt^, and results expressed as fold change.

## Results

### Abnormalities in mitochondrial metabolism are present in models of GSDIa

**Figure 1:**
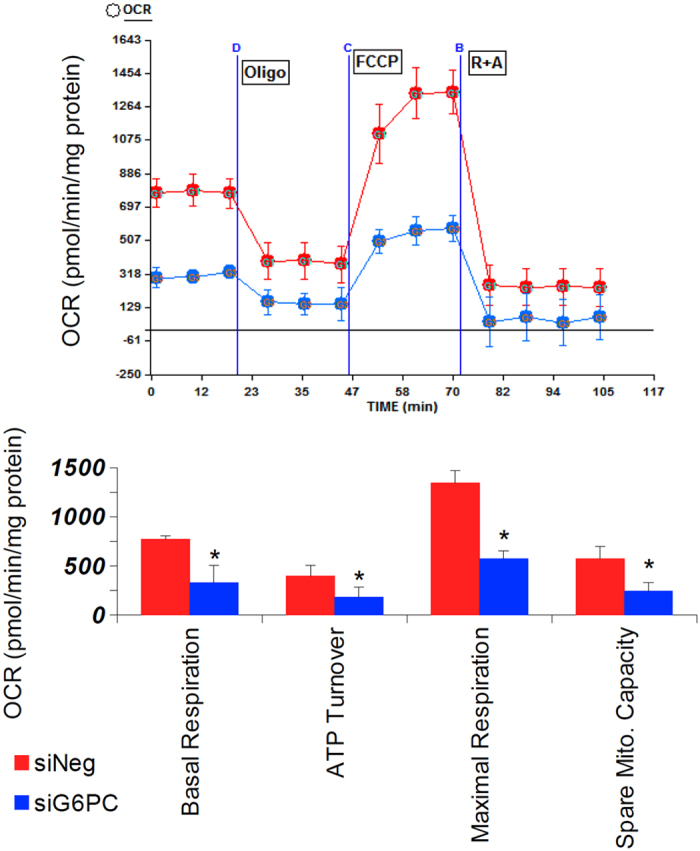
Mitochondrial respiration is impaired in G6PC KD versus control siRNA treated AML-12 cells. G6PC was knocked-down in AML-12 cells for 96 hours, then mitochondrial oximetry analysis was performed using a Seahorse XF24 mitochondrial flux analyser. Basal respiration, ATP turnover, Maximal Respiration, and Spare Mitochondrial capacity were measured/calculated as described in the methods section. Oxygen consumption was normalised to total cellular protein content. For all parts, n = 6, error bars represent SEM, *represents p < 0.05.

**Figure 2:**
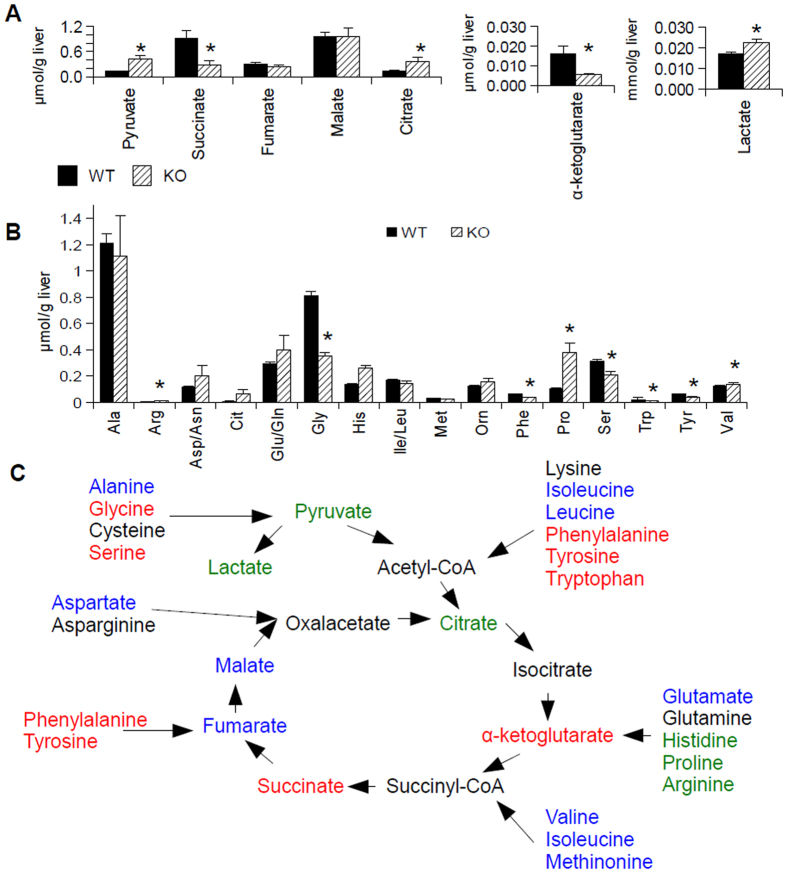
The tri-carboxylic acid cycle function is impaired in G6PC KO mice. (**A**) Organic acid analysis in the livers G6PC KO mice (KO, n = 6) compared to wild-type (WT, n = 10) mice. (**B**) Analysis of amino acid levels in the same samples. (**C**) Schematic of the changes of the species in the TCA cycle. Species in green were increased in G6PC KO mouse livers relative to WT livers, while those in red were decreased. Species in blue showed no significant change between groups, while those in black were not directly measured. For all parts, error bars represent SEM, *represents p < 0.05.

Since oxidative metabolism is an important function of mitochondria, we evaluated the function of the electron transport chain by performing oximetry on G6PC KD AML-12 cells using a Seahorse XF24 mitochondrial flux analyser, 96 hours after knock-down. We found a striking decrease in basal respiration, ATP turnover, maximal respiration, and spare mitochondrial capacity in the knock-down cells compared to cells treated with control siRNA, implying a loss of mitochondrial function in this disease (Fig. 1). Next, in order to determine the status of the upstream TCA cycle, metabolomic analysis of the levels of organic and amino acids in the livers of G6PC knock-out (KO) mice (in the fed state) was performed^28^. We observed an accumulation of citrate, lactate, and pyruvate and a decrease in the levels of succinate and α-ketoglutarate (Fig. 2A), though the results may be confounded by ischaemia, as the lactate to pyruvate ratio was high in the livers of both the wild-type and knock-out mice^29^. Species beyond α-ketoglutarate in the TCA cycle were present at similar levels as those found in wild-type mice, with the exception of succinate, which was decreased in the KO mice relative to the levels in wild-type mice (Fig. 2A). Analysis of amino acids levels showed a relative increase in the levels of histidine, proline, and arginine in the livers of the KO mice, with a relative decrease in glycine, phenylalanine, serine, tryptophan, and tyrosine levels in the KO mouse livers (Fig. 2B). One possible interpretation of this data is a block in the TCA cycle that occurred after citrate; however, pulse-chase analysis using tracers would be necessary to determine definitively the flux through each of these reactions. Interestingly, analysis of the same metabolites in G6PC KD AML-12 cells following 96 hours of knock-down did not entirely match the results seen in the *in vivo* system (Sup. Fig. 2). Although lactate was accumulated in both systems, the levels of the other organic acids did not show a statistically significant difference between the KD and wild-type cells (Suppl. Fig. 2A). Amino-acid analysis of the KD cells revealed relative decreases in the levels glycine, histidine, phenylalanine, proline, and valine (Suppl. Fig. 2B). This difference between the *in vivo* and cell culture systems implies that some of the changes in metabolite levels may not necessarily be cell-autonomous.

### Mitochondrial ultra-structure is abnormal in GSDIa

**Figure 3:**
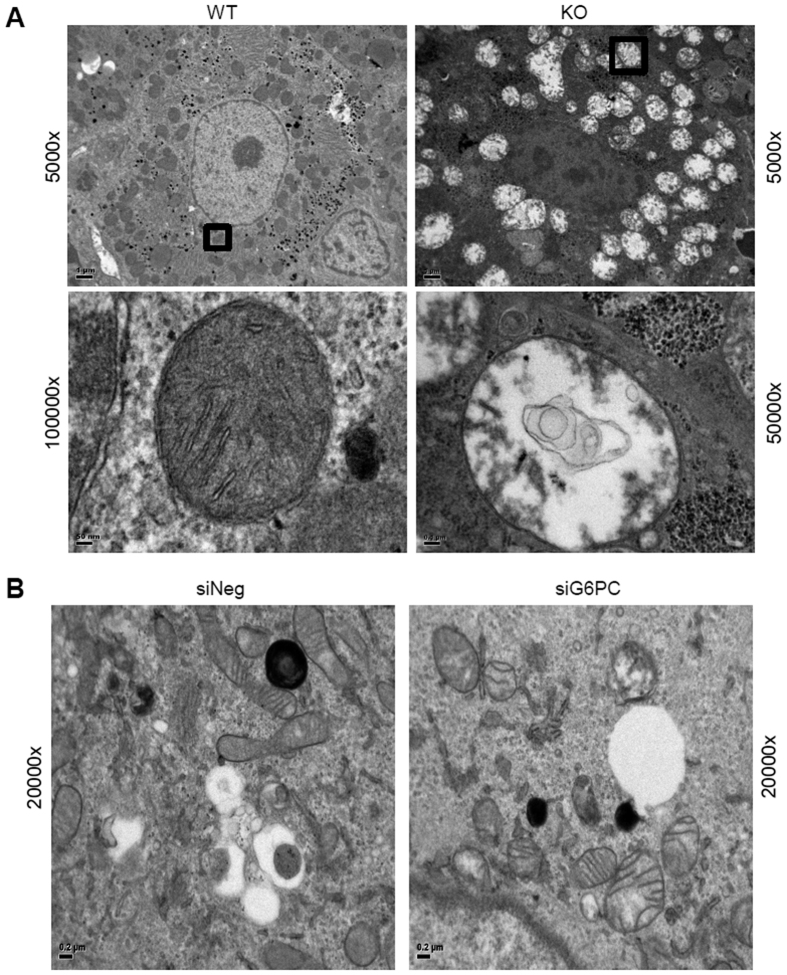
Mitochondrial morphology is deranged in the livers of G6PC KO mice and G6PC KD AML-12 cells (96 hour knockdown). Mitochondrial morphology was analysed in ultrathin sections of mouse liver (**A**) and AML-12 cells (**B**) by electron microscopy. Mitochondrial morphology in the wild-type mice and control cells were within normal limits, whereas in the KO mice and KD cells, the mitochondria were distended, and swollen, with effacement of the cristae, disruption of the mitochondrial membranes, and influx of cytoplasmic contents into the mitochondria. Representative images from the mouse liver (**A**) and KD cells (**B**) are displayed. Scale bars are marked on each image. Frames box areas shown at high magnification.

The structure of the mitochondria observed by electron microscopy (EM) in a cell is intimately linked to its functional status^30^. In healthy cells mitochondria have both inner and outer membranes, with the inner-membrane folded into structures known as “cristae”^30^, which are important for proper mitochondrial respiration^31^. When we examined the morphology of mitochondria in G6PC KO mice by EM, distended and swollen mitochondria were noted, as compared to normal mitochondria in the livers of their wild-type litter mates. Furthermore, mitochondria in the KO mice had numerous outer membrane ruptures, effacement of the cristae, as well as influx of cytoplasmic material into the mitochondria itself (Fig. 3A). Similar findings also were noted in AML-12 cells treated for 92 hours with siRNA targeting G6PC (G6PC KD cells), as compared to wild-type AML-12 cells (Fig. 3B). Thus, loss of G6PC not only altered mitochondrial function, but also led to large structural changes as well.

### Mitochondrial number and biogenesis, is decreased in GSDIa

**Figure 4:**
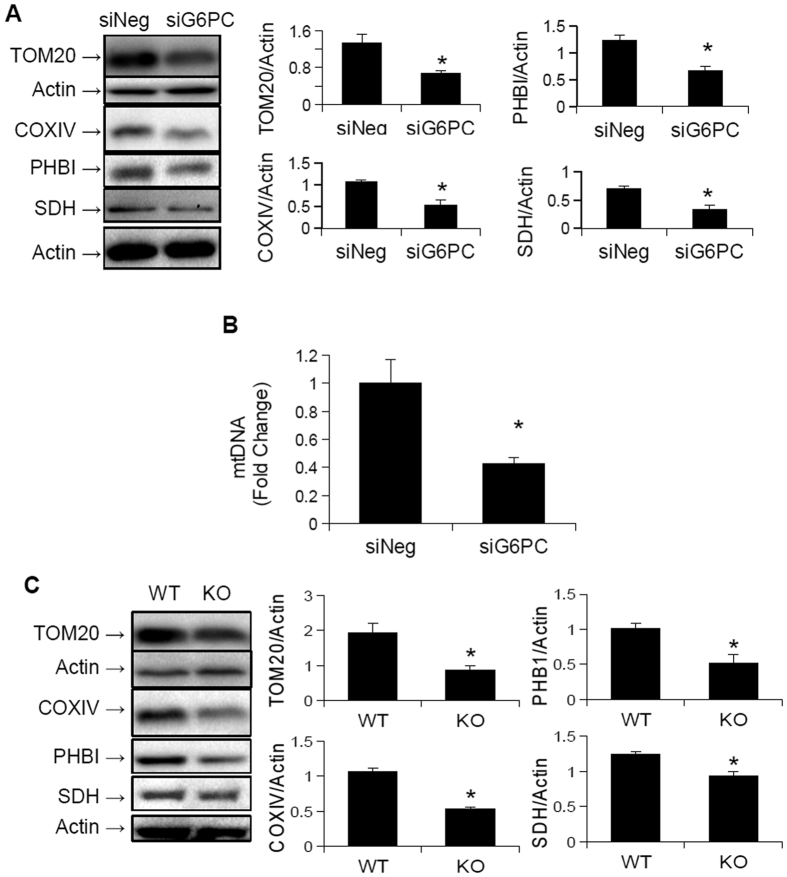
Mitochondrial content is reduced in GSDIa models. (**A**) The protein levels of various key mitochondrial proteins are reduced following G6PC KD for 96 hours in AML-12 cells (n = 3). (**B**) The copy number of mitochondrial DNA is also reduced following G6PC KD for 96 hours in AML-12 cells (n = 6). (**C**) G6PC KO mouse livers also show decreased levels of mitochondrial proteins (n = 3). *Represents p < 0.05, error bars represent SEM.

**Figure 5:**
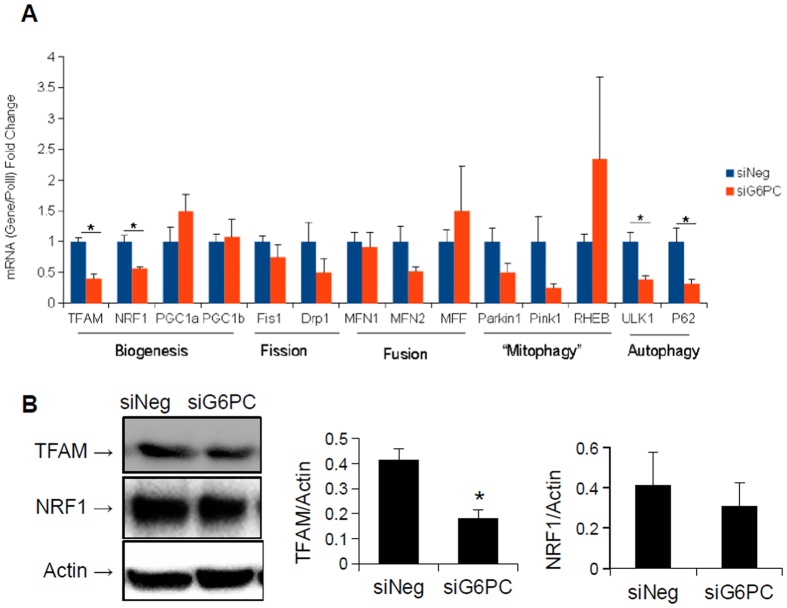
Key factors involved in mitochondrial biogenesis are reduced following G6PC KD for 96 hours in AML-12 cells. (**A**) Analysis of mRNA expression of genes involved in mitochondrial biogenesis, quality control and mitophagy shows a decrease in expression of genes related to mitochondrial biogenesis, with no change in expression of key fission and fusion genes. (n = 3). (**B**) Protein levels of TFAM are reduced following G6PC KD, however those of NRF1 show no significant change (n = 5). *Represents p < 0.05, error bars represent SEM.

**Figure 6:**
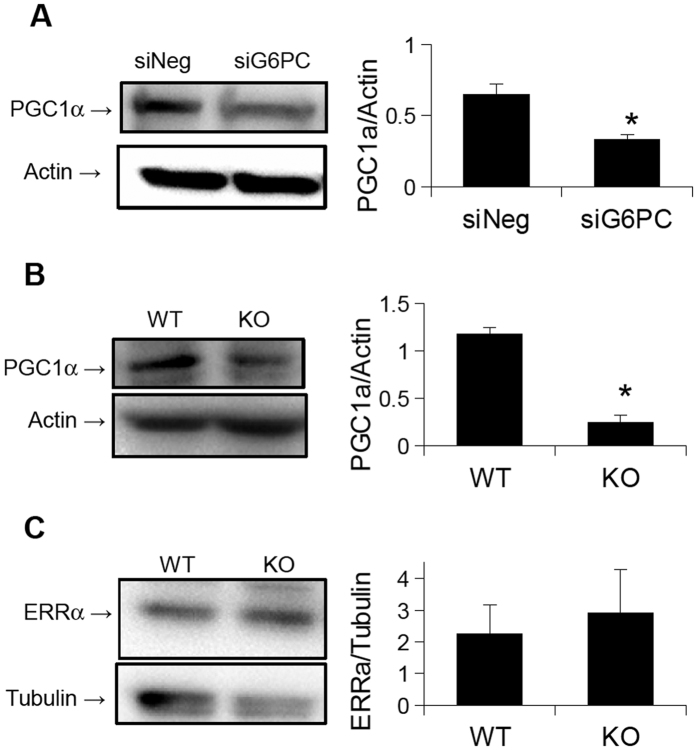
Protein levels of key transcription factors involved in mitochondrial biogenesis. (**A**) PGC1α levels are reduced in G6PC KD AML-12 cells (n = 5). (**B**) PGC1α levels are also reduced in G6PC KO mouse livers (n = 3). (**C**) Levels of ERRα are unchanged in G6PC KO mouse livers as compared to WT livers (n = 3). *Represents p < 0.05, error bars represent SEM.

Since we previously found that autophagy is impaired in GSDIa^16^, and autophagy of mitochondria (mitophagy) is crucial for the maintenance of mitochondrial function^32^ we hypothesised that the decrease in oxidative metabolism, as well as the altered mitochondrial ultra-structure resulted from a failure of mitophagy. Accordingly, we determined the levels of mitochondrial proteins in G6PC KO mouse livers and G6PC KD AML-12 cells since decreased mitophagy often is associated with an accumulation of mitochondrial proteins^33^. To our great surprise, mitochondrial protein levels, as well as mitochondrial genome copy number were reduced in G6PC KD cells as compared to wild-type cells (Fig. 4A,B). Furthermore, mitochondrial protein content was reduced in G6PC KO mice livers, compared to wild-type livers (Fig. 4C). Since these findings were not consistent with a decrease in mitophagy, we next hypothesised that the decreased levels of mitochondrial proteins were secondary to impaired mitochondrial biogenesis. We thus examined the expression of key genes involved in mitochondrial biogenesis in G6PC KD AML-12 cells. Interestingly, the mRNA levels of TFAM and NRF1, key regulators of mitochondrial biogenesis^17^, were both down-regulated in the KD cells(Fig. 5A). Analysis of the protein levels encoded by these genes showed a significant decrease in the levels of TFAM, and a trend towards a decrease in the levels of NRF1 (Fig. 5B). Of note, further analyses showed that there were no significant changes in the mRNA levels of key genes involved in fission or fusion, two important pathways for restoring mitochondrial function^20^. The expression of genes that were specifically involved in mitophagy also were not significantly changed (Fig. 5). However, the genes involved in general autophagy were down-regulated, as previously described^16^. Although the mRNA levels of PGC1α and PGC1β, two master regulators of mitochondrial biogenesis, were not affected by the knock-down of G6PC (Fig. 5), the protein level of PGC1α was reduced in G6PC KD AML-12 cells as well as G6PC KO mice (Fig. 6A,B). Interestingly, the levels of another key transcription factor involved in mitochondrial biogenesis, ERRα^34^, were unchanged in the livers of the KO mice (Fig. 6C). Thus the decreased oxidative respiration following loss of G6PC may also be due to a quantitative decrease in mitochondria, possibly secondary to decreased mitochondrial biogenesis. This failure in biogenesis occurred most likely due to reduced PGC1α protein levels, owing either to reduced translation or increased proteasomal degradation. Furthermore, there was no concomitant increase in the expression of genes involved in the fusion, fission, or mitophagy/autophagy pathways that might help compensate for, or repair, the decreased mitochondrial function.

### The mitochondrial apoptosis cascade is activated in GSDIa

**Figure 7:**
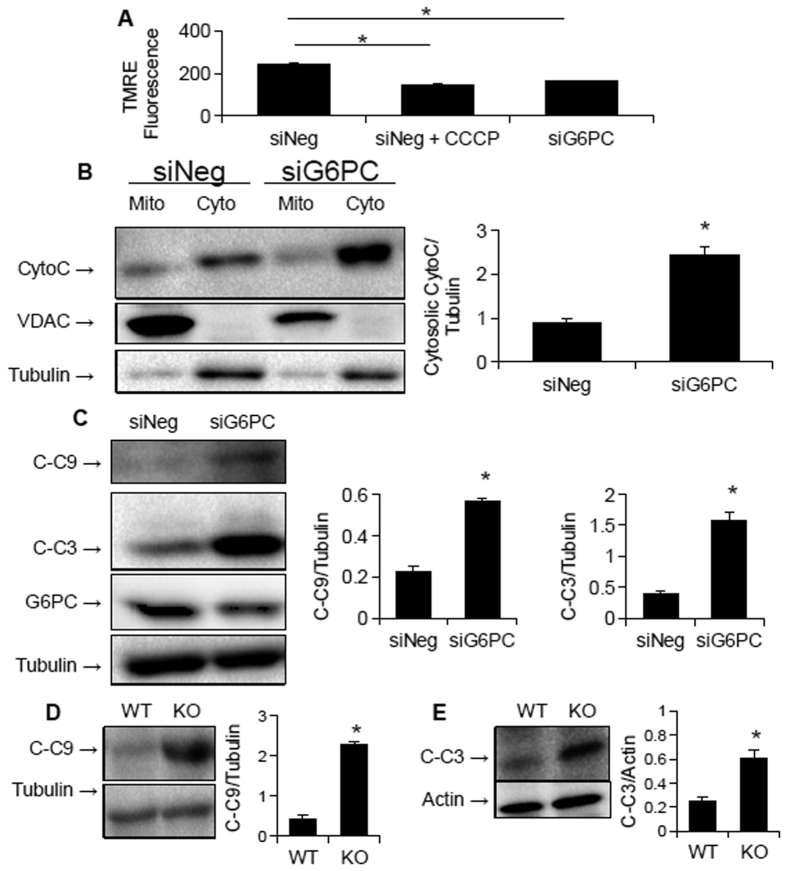
The mitochondrial apoptosis pathway is up-regulated in GSD1a. (**A**) Mitochondrial membrane potential is reduced in G6PC KD AML-12 cells 72 hours after knockdown. (**B**) Cytosolic cytochrome c is increased in G6PC KD AML-12 cells. (**C**) Caspase 9 and caspase 3 cleavage is increased in G6PC KD AML-12 cells. (**D**,**E**) Caspase 9 (**D**) and caspase 3 (**E**) cleavage is increasted in G6PC KO mice. For all parts except A, n = 3, error bars represent SEM, and *represents p < 0.05. For A, n = 6.

Since impaired mitochondrial function is linked to increased apoptosis^21^, we examined whether activation of the mitchondrial apoptosis cascade could explain the increased apoptosis seen in GSDIa^15^. To that end, we first examined mitochondrial membrane potential in G6PC KD cells 72 h after KD and found a striking decrease in membrane potential as measured by TMRE fluorescence (Fig. 7A). This loss of membrane potential was associated with translocation of cytochrome c into the cytoplasm (Fig. 7B), as well as cleavage and activation of caspase 9 (Fig. 7C), a specific marker of the mitochondrial apoptosis pathway^21^. The final step in the pathway, caspase 3 cleavage and activation, also was increased in the KD cells (Fig. 7C). Furthermore, increased cleavage of caspase 9 and 3 also was noted in the livers of G6PC KO mice (Fig. 7D,E).

### Potential causes for mitochondrial dysfunction

We then investigated whether decreased mitochondrial function was secondary to damage caused by increased ROS levels^35^. Surprisingly, ROS levels in G6PC KD AML-12 cells were not increased at 48, 72, or 96 hours following knock-down (Suppl. Fig. 3). We also hypothesised that the decrease in mitochondrial function may be secondary to increased mitochondrial protein acetylation, as acetyl-CoA levels were reported to be high in GSDIa^5^, and increased mitochondrial protein acetylation has been linked to impaired mitochondrial function^36^. However, we did not observe any increase in mitochondrial protein acetylation in G6PC KD AML-12 cells 96 hours after knock-down (Suppl. Fig. 4).

## Discussion

In this paper, mitochondrial function, structure, and number all were investigated in *in vitro* and *in vivo* models of GSDIa. Mitochondrial oxidative metabolism was impaired (Fig. 1), and levels of TCA cycle intermediates were changed both *in vitro* and *in vivo* (Fig. 2). These findings were associated with changes in mitochondrial ultrastructure (Fig. 3), decreases in mitochondrial content (Fig. 4), impaired mitochondrial biogenesis (Figs 5 and 6), and activation of the mitochondrial apoptosis pathway (Fig. 7). Taken together, these findings point to a previously unknown role for mitochondrial damage in the pathogenesis of GSDIa. Although changes in mitochondrial structure in this disease were noted in one study^25^, most studies of this disorder have focused primarily on biochemical abnormalities^3^, and have overlooked the functional status of organelles such as mitochondria. Thus, these findings may help explain some previously described features of GSDIa, such as the increased hepatocellular apoptosis that occurs in this disorder^15^.

The cause of the mitochondrial dysfunction is not known. Although it is possible that the decrease in mitochondrial oxidation could solely be due to decreased mitochondrial content, it is likely that a qualitative defect in mitochondrial function also exists, since ultra-structural analysis of both KO mice and KD AML-12 cells showed large numbers of damaged mitochondria. The changes in mitochondrial function do not appear to be due increased ROS production and oxidative damage, nor due to altered acetylation of mitochondrial proteins. However, the change in membrane potential suggests that defects in membrane structure, perhaps by altered or imbalanced incorporation of fatty acids into the mitochondrial membrane may be involved. Gluctoxicity^37^, or lipotoxicity due to lipid species such as saturated free fatty acids^38^ or ceramides^39^ also may be mediators of mitochondrial damage in this disease. Thus, it is possible that the excessive intracellular carbohydrates or lipids in this disease may lead to the mitchondrial dysfunction. Although decreased mitophagy may be present in this system, we did not observe an accumulation of mitochondrial proteins in either cell culture or *in vivo* models to support this hypothesis. Moreover, decreased mitophagy cannot explain the rapid deterioration in mitochondrial function observed in our cell culture experiments. In this connection, previous work has shown that mitochondrial function is maintained 90 days after deletion of ATG7 in mouse liver, despite changes in morphology^40^. Likewise, the cause of the down-regulation of PGC1α protein levels is not yet known. Since its transcription was not reduced, translational or post-translational effects are likely to be involved in the reduction of PGC1α levels. Additionally, our group recently showed that decreased autophagy is associated with decreased protein levels of PGC1α^41^, and since autophagy is decreased in GSDIa, it is possible that a similar mechanism may be involved here^16^.

The recent epidemic of non-alcoholic fatty liver disease (NAFLD) has been attributed, in part, to excess carbohydrate intake. Since G6PC deficiency prevents gluconeogenesis and causes accumulation of excess intracellular glucose-6-phosphate, it is a genetic model of glucose toxicity. Recent studies have suggested that mitochondrial dysfunction occurs in NAFLD, and may play an important role in its pathogenesis, as well as in the development of insulin resistance^42^. Indeed, mice that are heterozygous for a defect in the mitochondrial trifunctional protein develop hepatic steatosis and insulin resistance^43^. Thus, the progression of mitochondrial damage and accompanying metabolic changes in GSDIa may shed light on similar changes that occur during different stages of NAFLD.

Although the precise mechanism for the mitochondrial dysfunction in GSDIa is not well understood, it is clear that there is a severe dysfunction. Currently, liver transplantation^44^ or gene therapy^13^ seem to offer the best possibilities for definitive cure of GSDIa. However, issues with these therapies suggest that other adjunct treatments of GSDIa are needed. Primary mitochondrial disorders are treated by such means as l-carnitine supplementation, and/or elimination of dietary mitochondrial toxins, and these approaches might be warranted in GSDIa^45^. Even if gene therapy is successful, increased apoptosis and hepatocellular turnover may still impair its efficacy^46^, and thus treatments to improve mitochondrial function and prevent apoptosis may still be beneficial. Understanding the role of mitochondria in of the pathophysiology of GSDIa may lead to novel therapies to improve outcomes in patients with this disease as well as other conditions of lipid over-accumulation such as NAFLD.

## Additional Information

**How to cite this article:** Farah, B. L. *et al*. Hepatic mitochondrial dysfunction is a feature of Glycogen Storage Disease Type Ia (GSDIa). *Sci. Rep.* **7**, 44408; doi: 10.1038/srep44408 (2017).

**Publisher's note:** Springer Nature remains neutral with regard to jurisdictional claims in published maps and institutional affiliations.

## Supplementary Material

Supplementary Information

## Figures and Tables

**Table 1:** Primer sequences for real-time PCR of genes related to GSDIa and mitochondrial function.

Gene	Forward Sequence	Reverse Sequence
PolII	CGGTTGAATCTTAGTGTGAC	ATAGCCAACTCTTGGATCTC
Tfam	GAGCGTGCTAAAAGCACTGG	GACAAGACTGATAGACGAGGGG
Nrf1	CTGCAGGTCCTGTGGGAAT	GAAGCTGAGCCTGGGTCATT
Pgc1α	GTCATGTGACTGGGGACTGT	AACCAGAGCAGCACACTCTAT
Pgc1β	CTCTGACACGCAGGGTGG	GAAGAGCTCGGAGTCATCGG
Fis1	TTAGTGTGAGGCTTTCAGGGG	CAGTCCATCTTTCTTCATGGCCT
Drp1	GCCTCAGATCGTCGTAGTGG	TTCCATGTGGCAGGGTCATT
Mfn1	ATTGCCACAAGCTGTGTTCG	GTTTTCCAAATCACGCCCCC
Mfn2	ACCAGCTAGAAACTTCTCCTCT	CTTCGCTGATACCCCTGACC
Mff	TCACATTTGGTGAGTGGGGC	TTTTCCGGGACCCTCATTCG
Parkin	GCAGCCAGAGGTCCAGTTAAA	GAGGGTTGCTTGTTTGCAGG
Pink1	TTGCAATGCCGCTGTGTATG	TGGAGGAACCTGCCGAGATA
Rheb	CGGTCTGTGGGAAAGTCCTC	ATATTCATCCTGCCCCGCTG
Ulk1	GCTGTGTAGTGGGAGCTCAT	TGGGGAGAAGGTGTGTAGGG
Sqstm1/p62	AGCTGCCCTCAGCCCTCTA	GGCTTCTCTTCCCTCCATGTT

**Table 2:** Metabolic parameters for mice used in these studies.

	G6PC^+/+^	G6PC^−/−^
Body Weight (g)	7.73 + 1.41	4.45 + 0.26
Liver Weight (g)	0.268 + 0.464	0.416 + 0.029
Blood Glucose (mg/dl)	203.5 + 47.18	≤20
Hepatic Triglyceride (mg/g protein)	35.78 + 3.72	307.62 + 41.62
Hepatic Glycogen (mmol glucose/g protein)	0.18 + 0.03	7.02 + 1.76

Triglyceride and Glycogen data is from previously published work using these mice^16^. Values are expressed as mean + SEM.
