# Supplementary material for: Hepatic mitochondrial dysfunction is a feature of Glycogen Storage Disease Type Ia (GSDIa)
Source: Sci Rep. 2017 Mar 20;7:44408. doi: 10.1038/srep44408 (PMC5357851; doi:10.1038/srep44408)
Supplement: Supplementary Information [file srep44408-s1.pdf]

Hepatic mitochondrial dysfunction is a feature of Glycogen Storage Disease Type Ia (GSDIa)

Benjamin L. Farah[1], Rohit A. Sinha[1], Yajun Wu[2], Brijesh K. Singh[1], Andrea Lim[1], Masahiro Hirayama[3], Dustin J. Landau[5], Boon-Huat Bay[2], Dwight D. Koeberl[4,5], Paul M. Yen*[1,6]

Supplementary Figure Legends

Supplementary Figure 1: Typical protein levels of G6PC in AML-12 cells following 96 HR siRNA knock-down. n=5, error bars represent SEM, * represents $p<0.05$

Supplementary Figure 2: Metabolomic analysis of organic acids and amino-acids in G6PC knock-down AML-12 cells. Organic acid **(A)** and amino acid **(B)** levels in AML-12 knock-down cells. Cells were grown for 96 hours following knock-down, then harvested for metabolomic analysis. Metabolite levels are normalized to protein concentration from the same sample. For all parts, n= 5, error bars represent SEM, * represents $p<0.05$.

Supplementary Figure 3: Mitochondrial ROS production is reduced following G6PC KD. G6PC was knocked-down in AML-12 cells, and 48, 72, and 96 hours after knock-down cellular ROS levels were assayed by measuring DCFDA fluorescence. tBHP was used as a positive control for increased ROS levels. n=6, error bars represent SEM, * represents $p<0.05$

Supplementary Figure 4: There is no increase in mitochondrial protein acetylation following G6PC KD A.) Purity analysis of mitochondrial and cytosolic fractions of AML-12 cells following G6PC KD and mitochondrial fractionation. B.) Levels of lysine-acetylated proteins in the mitochondrial fractions of WT and G6PC KD cells. For all parts, n=3, error bars represent SEM, * represents $p<0.05$.

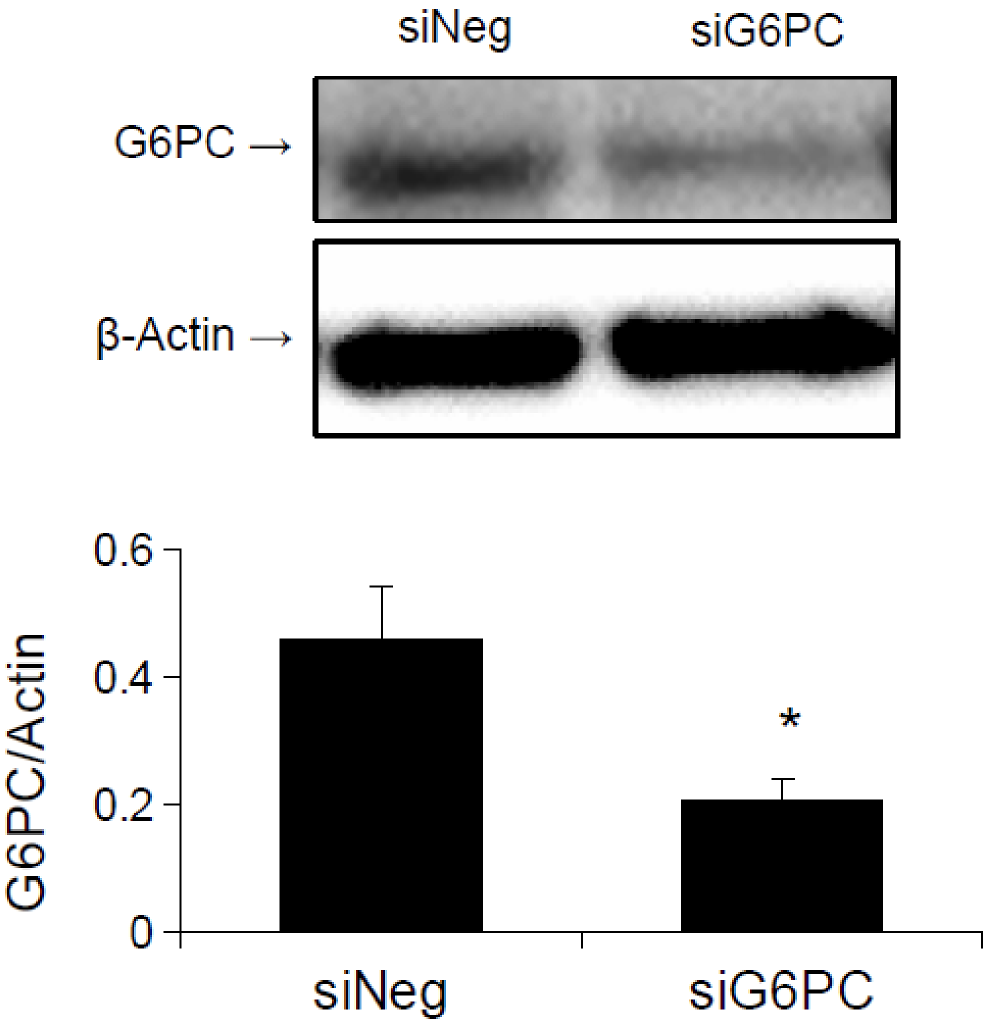

Supplementary Figure 1

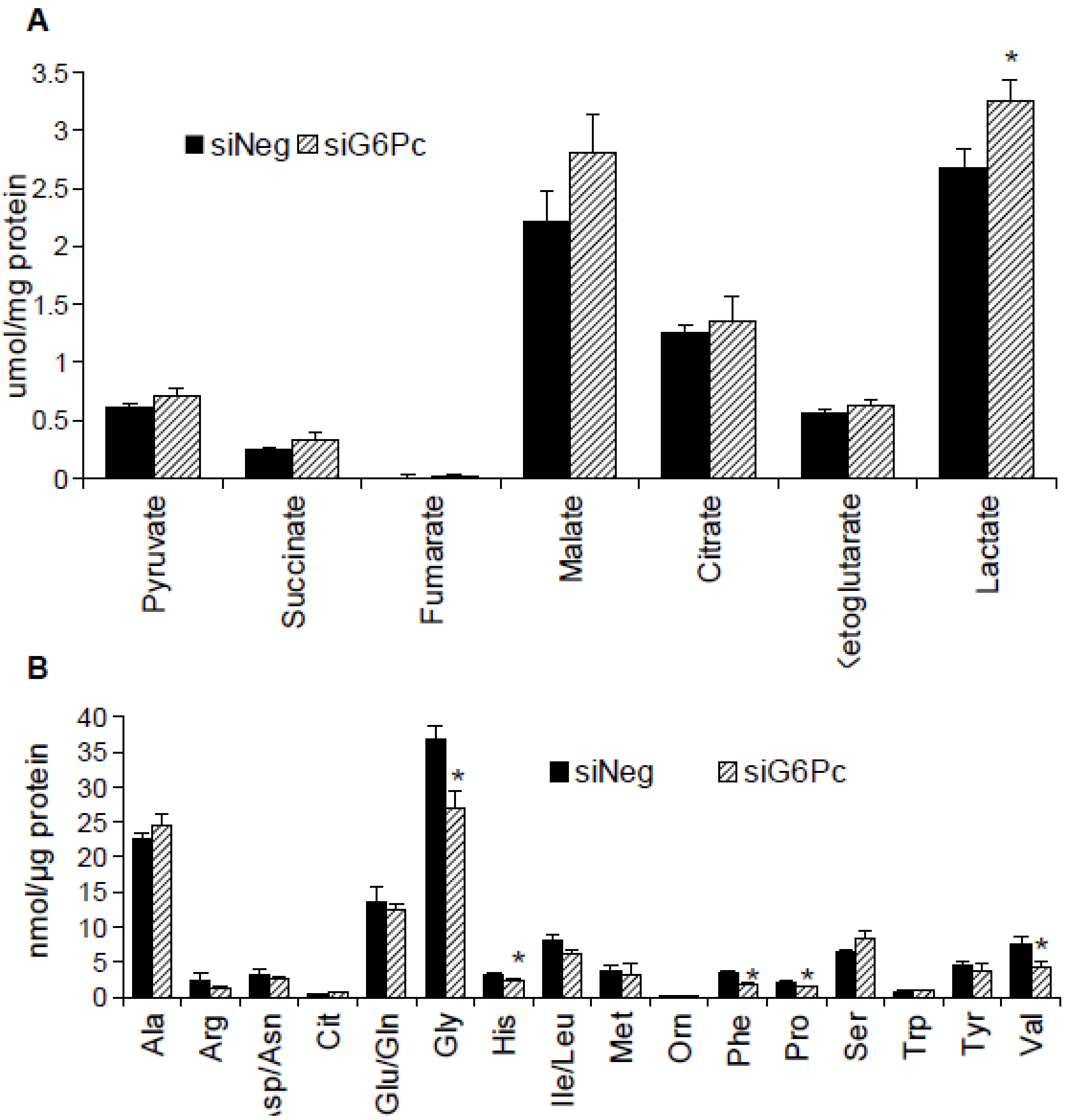

Supplementary Figure 2

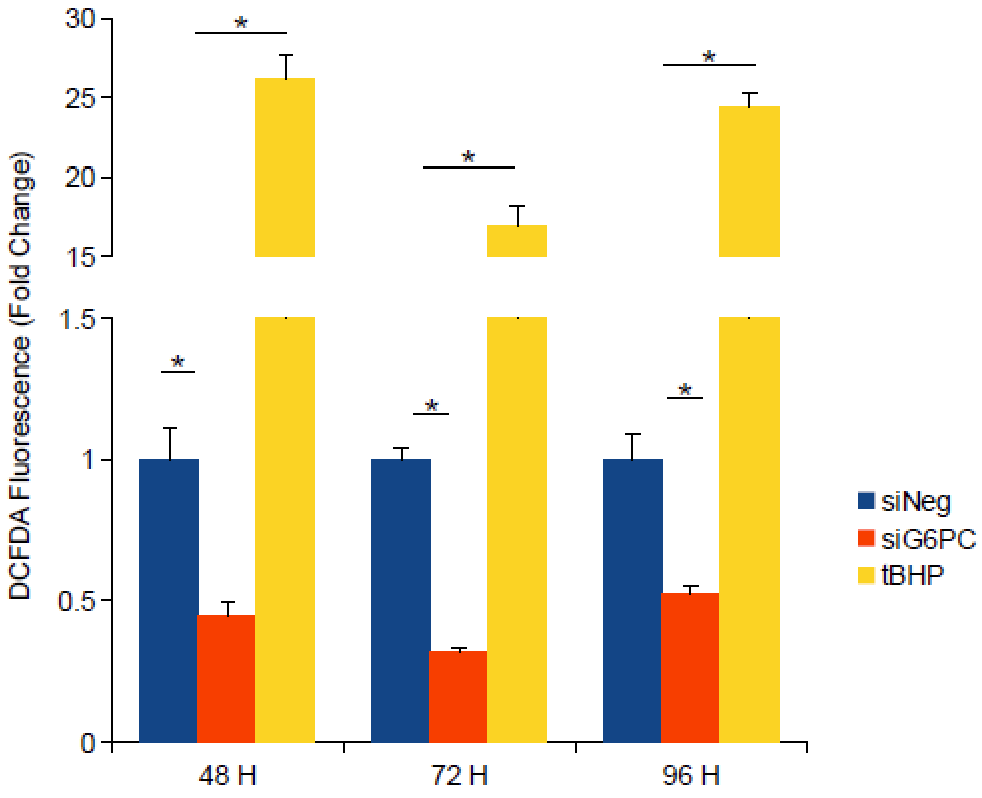

Supplementary Figure 3

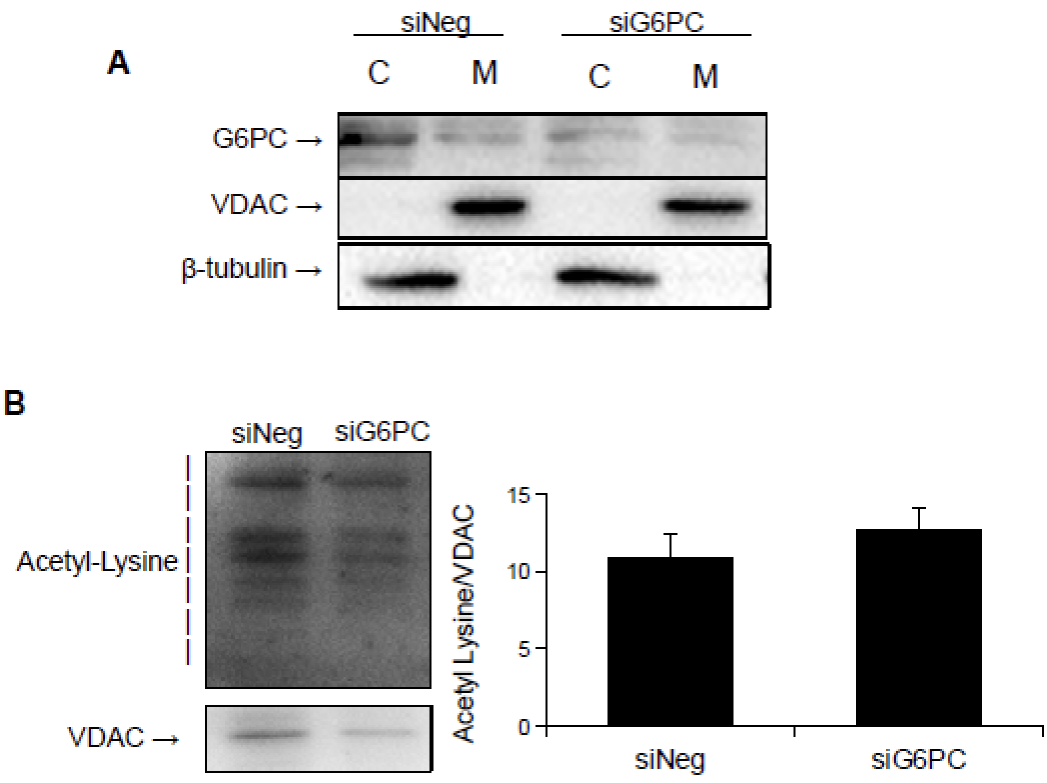

Supplementary Figure 4
